# Transient Acquired von Willebrand Disease With a Type 2B Phenotype: Recognizing the Diagnostic Challenges

**DOI:** 10.7759/cureus.83470

**Published:** 2025-05-04

**Authors:** Sumayya Aslam, Lila Nguyen, Laura Rabichow, Janet Dinh, Jesse C Qiao

**Affiliations:** 1 Pathology and Laboratory Medicine, University of California (UC) Irvine, Irvine, USA; 2 Hematology Laboratory, University of California (UCI) Health, Orange, USA

**Keywords:** bleeding, diffuse large b cell lymphoma (dlbcl), platelet aggregation, thrombocytopenia, von willebrand disease

## Abstract

Acquired von Willebrand disease (vWD) may arise secondary to autoimmune conditions, hematologic malignancies, or valvular heart dysfunction. It should be included in the differential diagnosis of any acquired bleeding disorder. Most cases resemble Type 1 or Type 2A inherited vWD, typically presenting with variable decreases in von Willebrand antigen and activity. However, clinicians should exercise caution when interpreting von Willebrand panels to rule out acquired vWD, as acute phase reactants - associated with inflammation and malignancy - can artificially elevate and normalize circulating von Willebrand factor (vWF) and factor VIII levels.

We present a rare case of acquired vWD with a Type 2B phenotype on platelet aggregation studies, temporally associated with diffuse large B-cell lymphoma (DLBCL). vWF and multimer assays were non-contributory, likely due to elevated acute phase reactants at the time of diagnosis, evidenced by increased factor VIII activity and falsely elevated von Willebrand antigen and activity levels. The patient's bleeding symptoms resolved following lymphoma treatment and remission. Platelet aggregation normalized, factor VIII levels returned to baseline, and von Willebrand activity stabilized.

To the best of our knowledge, this case is the first to describe an association between acquired vWD with a Type 2B phenotype and DLBCL, expanding the range of hematologic malignancies linked to this rare presentation. We emphasize diagnostic challenges, particularly the role of acute-phase reactants in obscuring von Willebrand panel interpretations.

## Introduction

Acquired von Willebrand disease (vWD) should be considered in elderly patients with new-onset bleeding or bruising, particularly when there is no prior personal or family history of bleeding. While the 2021 American Society of Hematology (ASH) guidelines provide a framework for evaluating vWD, they presume patients are in a steady state of health - a condition not met in cases of acquired vWD [1]. Acquired vWD can present with mucocutaneous bleeding patterns similar to those of mild to moderate inherited vWD, particularly Types 1 and 2A [2]. This condition arises through several distinct mechanisms: adsorption of von Willebrand factor (vWF) onto abnormal cell surfaces (e.g., in lymphoproliferative disorders), neutralization by autoantibodies, and increased proteolysis due to shear stress (e.g., in patients with valvular heart disease or mechanical circulatory devices) [3]. A rarer mechanism involves acquired 2B-like phenotypes, typically associated with monoclonal gammopathies and detected via platelet aggregation testing [4-6]. Although the presence of paraproteins can support the diagnosis, they are not required; structural or functional disruption of vWF may occur independently [7].

Interpretation of vWF panels is particularly challenging during inflammatory states or acute illness. As vWF and factor VIII are acute-phase reactants, their levels may be elevated - masking an underlying deficiency or dysfunction. This is also true during physiologic stressors such as pregnancy and the postpartum period. In such cases, the standard diagnostic panel for inherited vWD-vWF antigen, vWF activity, and factor VIII levels - may yield misleading results [8,9]. Age-related increases in vWF and factor VIII further complicate interpretation in older adults [10]. Moreover, multimer analysis may appear deceptively normal despite clinically significant dysfunction in the presence of acute-phase reactants. As a result, “normal” laboratory values cannot reliably exclude acquired vWD when clinical suspicion remains high [11-14]. In select cases, the von Willebrand propeptide assay may help demonstrate increased vWF turnover, supporting the diagnosis [15].

Clinicians and laboratorians must recognize the limitations of standard von Willebrand assays and the potential for false-negative or seemingly “normal” patterns. This case report highlights the diagnostic complexity of acquired vWD in a patient with clinically significant bleeding and an unusual platelet aggregation phenotype. We discuss the pitfalls of conventional testing and the role of supplementary assays in confirming the diagnosis.

## Case presentation

**Figure 1:**
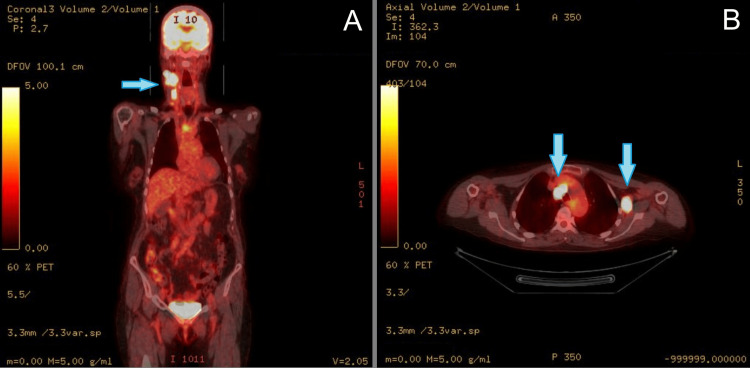
Whole-body FDG PET/CT demonstrating abnormal radiotracer uptake in the right neck, mediastinum, and left shoulder region. Representative FDG PET/CT images at the time of diagnosis demonstrate increased tracer uptake in the enlarged right cervical lymph nodes (Panel A, coronal section), prominent mediastinal lymph nodes (Panel B, transverse section), and enlarged left axillary lymph nodes (Panel B, transverse section), compatible with B-cell lymphoma. Abnormalities are denoted with blue arrows above. The image has been fully de-identified in accordance with the institution's review board (IRB) policy, which does not require patient consent for the use of de-identified clinical data in publications. FDG PET/CT, fluorodeoxyglucose positron emission tomography/computed tomography

A 71-year-old female with a past medical history of hypertension, hysterectomy, appendectomy, and an unremarkable family history presented with rapid, painless neck swelling over several weeks. Following a cervical lymph node biopsy, she was diagnosed with high-grade diffuse large B-cell lymphoma (DLBCL) with a “double hit” phenotype (mutations in both MYC and BCL2). A staging bone marrow biopsy showed no evidence of neoplastic involvement. A positron emission tomography (PET) scan demonstrated increased radiotracer uptake of several cervical and thoracic lymph nodes (Figure 1), but did not show advanced disease in the lower thoracoabdominal areas.

Shortly after diagnosis, the patient began chemotherapy with rituximab, cyclophosphamide, doxorubicin, vincristine, and prednisone (R-CHOP), initiated approximately one to two weeks after the initial lymph node biopsy. Around the same time, she developed patchy subconjunctival hemorrhages and new-onset symptoms, including erythematous papules and easy bruising with routine daily activities.

To investigate the bleeding, the treating hematologist referred the patient for additional laboratory testing. Her hemoglobin was low at 9.8 g/dL (normal: 12.5-17.0 g/dL), and her initial platelet count was decreased at 95 k/uL (normal: 150-450 k/uL). A basic metabolic panel (sodium, potassium, chloride, bicarbonate, anion gap, calcium, blood urea nitrogen, creatinine, and glucose) was within normal limits. Total protein was mildly decreased at 5.8 g/dL (normal: 6.0-8.5 g/dL). The patient’s blood type was O positive. Prothrombin time (PT) was 10.2 seconds (normal: 9.1-12.5 seconds), and activated partial thromboplastin time (aPTT) was 28.5 seconds (normal: 24.7-36.2 seconds). Serum protein electrophoresis did not reveal abnormal paraproteins. Platelet function analysis (PFA-100) showed a borderline normal collagen-epinephrine clotting time of 134 seconds (normal: <135 seconds), and a collagen-ADP clotting time of 101 seconds (normal: <110 seconds). A von Willebrand panel revealed an antigen (vWF:Ag) of 138% (normal: 50-160%), activity (vWF:RCo) of 89% (normal: 50-158%), and a factor VIII activity (FVIII:C) elevated at 214% (normal: 60-150%). The von Willebrand activity-to-antigen ratio was mildly decreased to 0.6 (normal: 0.7 or higher). von Willebrand multimer analysis, sent to a reference laboratory, showed no multimeric loss.

**Figure 2:**
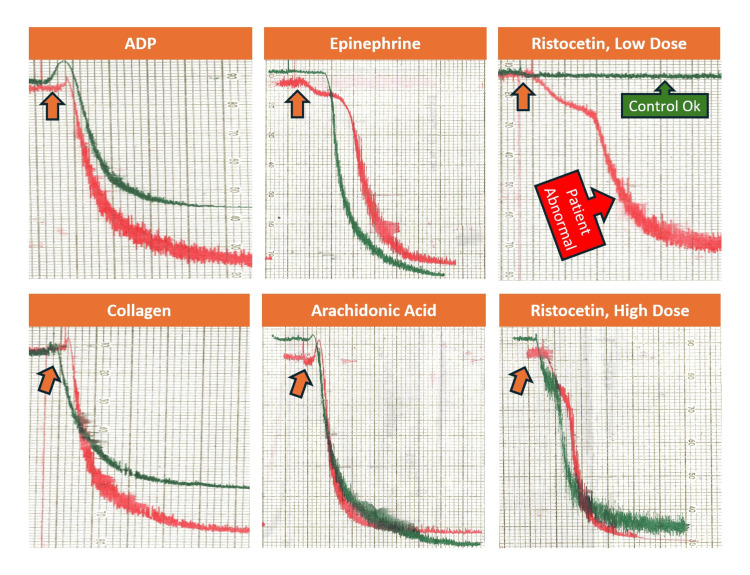
Light transmission platelet aggregometry findings at the initial presentation of bleeding while undergoing chemotherapy. The test was performed using the patient’s platelet-rich plasma in response to the addition of the following agonists: ADP 10 μg/mL (top left), collagen 2 μg/mL (bottom left), epinephrine 5 μg/mL (top center), arachidonic acid 0.5 μg/mL (bottom center), ristocetin - low dose (0.4 μg/mL, top right), and ristocetin - high dose (1.2 μg/mL, bottom right). The upward arrow approximately indicates the time of agonist addition for each aggregation assay. Red and green lines represent patient and normal donor control reactions, respectively. On each of the six subgraphs: the X-axis reflects the proportional time elapsed since each agonist was added to the patient’s platelet-rich plasma (each subset represents approximately five to six minutes of reaction time); the Y-axis reflects the proportional percentage of light transmission (top of scale: 0% light transmission denoting no platelet aggregation; bottom of scale: 100% light transmission denoting full platelet aggregation). ADP, adenosine diphosphate

With “normal” von Willebrand assays non-contributory to our case, platelet aggregation studies were subsequently requested to investigate a potential acquired platelet function disorder. Unexpectedly, a hyperactive (abnormal) response was noted with the agonist low-dose ristocetin (0.4 µg/mL), while the remainder of the platelet aggregation studies were unremarkable. While this characteristic platelet aggregation pattern is pathognomonic for inherited Type 2B or Platelet-Type (PT) vWD, this finding was highly unexpected with our clinical presentation of acquired bleeding. Platelet aggregation testing was repeated on two separate samples by two different laboratory technologists, and the results were verified to be accurate. Figure 2 depicts platelet aggregation morphology.

The patient did not require specific treatment for bleeding, as her symptoms remained mild and self-limited with observation. Following the completion of chemotherapy for DLBCL, her bleeding and bruising symptoms gradually improved and ultimately resolved. She remained in remission and symptom-free. Although genetic testing for VWF was considered, the hematologist opted first to repeat the previously performed tests. Repeat complete blood count (CBC), PT/aPTT, PFA-100, von Willebrand assays, and platelet aggregation studies were all unremarkable. Her von Willebrand panel showed normalization of factor VIII activity to 130%, von Willebrand antigen to 118%, von Willebrand activity to 106%, and complete resolution of thrombocytopenia. Table 1 summarizes the laboratory findings before and after treatment for DLBCL.

**Table 1:** Comparison of results at DLBCL presentation and post-treatment. (L), results below the lower reference range limit; (H), results above the upper reference range limit. PFA-100, platelet function analyzer; Col/EPI, collagen-epinephrine agonists; Col/ADP, collagen-adenosine diphosphate agonists; vW, von Willebrand; RCo, ristocetin cofactor; PT, prothrombin time; aPTT, activated partial thromboplastin time; s, seconds; g, grams; dL, deciliter; µL, microliter; %, percent; N/A, not applicable

Laboratory/Clinical	At Diagnosis	At Remission	Reference Ranges
Clinical Bleeding/Bruising?	Yes	No	N/A
Hemoglobin	9.8 g/dL (L)	13.5 g/dL	12.5-18.0 g/dL
Platelet Count	95 k/µL (L)	169 k/µL	150-400 k/µL
Total protein	5.8 g/dL (L)	6.3 g/dL	6.0-8.5 g/dL
PFA-100 Col/EPI	134 s	126 s	<135 s
PFA-100 Col/ADP	101 s	74 s	<110 s
vW Antigen	138%	118%	50-160%
vW RCo Activity	89%	106%	50-158%
vW Activity to Antigen Ratio	0.6 (L)	0.9	≥0.7
Factor VIII Activity	214% (H)	130%	60-150%
von Willebrand Multimers	No loss	No loss	Normal
PT	10.2 s	10.3 s	9.1-12.5 s
aPTT	28.5 s	33.9 s	24.7-36.2 s

## Discussion

**Figure 3:**
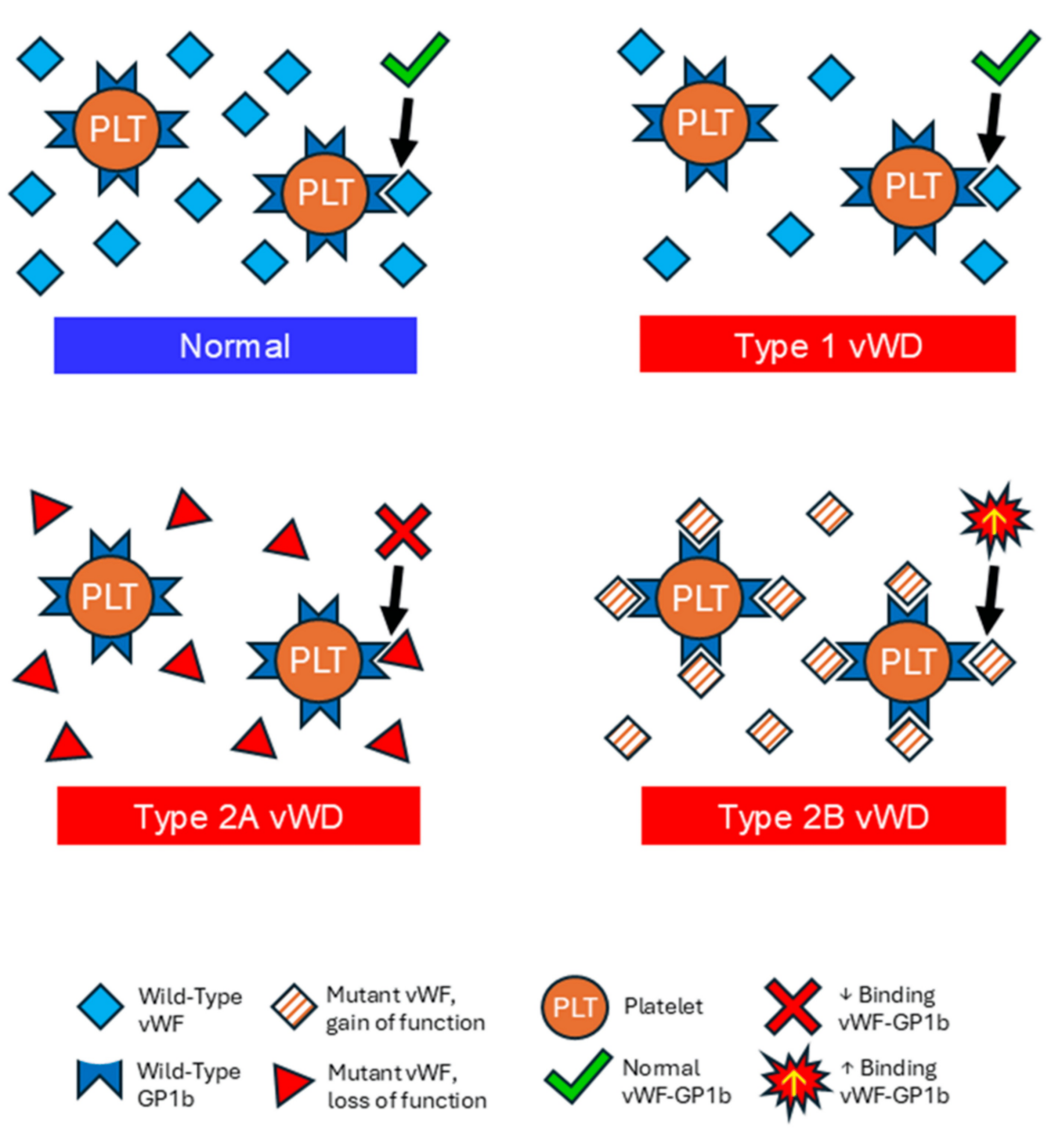
Interactions between plasma von Willebrand and platelet receptors. This figure compares interactions between plasma vWF and the platelet GPIb receptor in hemostasis and associated bleeding disorders (von Willebrand disease). Symbol keys are provided at the bottom of the figure. Top left: Normal interactions between plasma vWF and platelet GPIb binding; Top right: decreased plasma vWF with normal GPIb binding, in Type 1 vWD; Middle left: Mutation in vWF that results in multimer loss leading to decreased GPIb binding, in Type 2A vWD; Bottom right: Gain-of-function mutation of vWF that results in increased vWF-GPIb binding leading to premature clearance, in Type 2B vWD. vWF, von Willebrand factor; vWD, von Willebrand disease; GPIb, platelet glycoprotein Ib receptor

Given the normalization of prior platelet aggregation abnormalities, our clinical and laboratory findings support a diagnosis of transient acquired vWD with a Type 2B phenotype on platelet aggregation, temporally associated with DLBCL. “Normal” von Willebrand antigen and activity levels should still be carefully considered in the setting of an acquired bleeding diathesis with bruising. For illustrative purposes, a visual depiction of vWF interactions with platelet receptors in Types 1, 2A, and 2B vWD phenotypes, as well as in normal physiology, is presented in Figure 3.

In inherited Type 2B vWD, there is typically a decrease in both antigen and activity levels, along with an activity-to-antigen ratio of less than 0.7. Although our ratio was less than 0.7 - compatible with a Type 2 pattern - values that fall within the “normal” reference range may mislead clinicians into assuming von Willebrand function is intact if not critically examined in conjunction with the clinical context. Although serum protein electrophoresis was not performed to detect paraproteins or antibodies that might bind to vWF, the patient's low serum protein and normal staging bone marrow biopsy make this scenario unlikely to explain the acquired vWD.

Platelet aggregation morphology provided key evidence supporting acquired vWD through an abnormality in ristocetin-induced platelet aggregation, with the disappearance of this abnormality following treatment and remission of DLBCL. One of the main challenges we faced was reconciling a morphology classically associated with an inherited bleeding phenotype (Type 2B vWD) with our patient’s acquired bleeding presentation. 

Inherited Type 2B vWD is caused by gain-of-function point mutations in the vWF gene A1 domain [16]. These mutations increase vWF binding affinity to platelet GPIb, resulting in premature plasma clearance of vWF, variable degrees of thrombocytopenia, mild to moderate bleeding manifestations, and a hyperactive response to low-dose ristocetin-induced platelet aggregation [16]. While Type 2B vWD may lead to the loss of high-molecular-weight multimers, this is not always observed; a subset of patients presents with normal multimer patterns [17], especially when confounded by acute-phase reactants or inflammation that may “normalize” or mask vWF deficiencies.

Dismissing this Type 2B pattern abnormality as incidental or erroneous, particularly in the face of a discordant clinical presentation, would not be appropriate for patient care. Special coagulation testing was prompted by the patient’s active bleeding and bruising. Furthermore, the low-dose ristocetin platelet aggregation testing was performed and repeated with quality assurance by two separate coagulation specialist technologists, and was verified by the interpreting pathologist.

Genetic testing of known vWF mutations can help distinguish between inherited and acquired vWD when differentiation is unclear, or when ruling out inherited disease is necessary [18]. While genetic testing may provide a definitive diagnosis, it is limited to known mutations in vWF and associated platelet GPIBA (glycoprotein Ib alpha) genes. Cost considerations and the need for appropriate patient counseling must also be factored in when considering genetic testing. In our case - with no family history of bleeding, normal repeat testing, and resolution of clinical bleeding - genetic testing would not have been necessary, though it may offer utility in certain scenarios.

**Figure 4:**
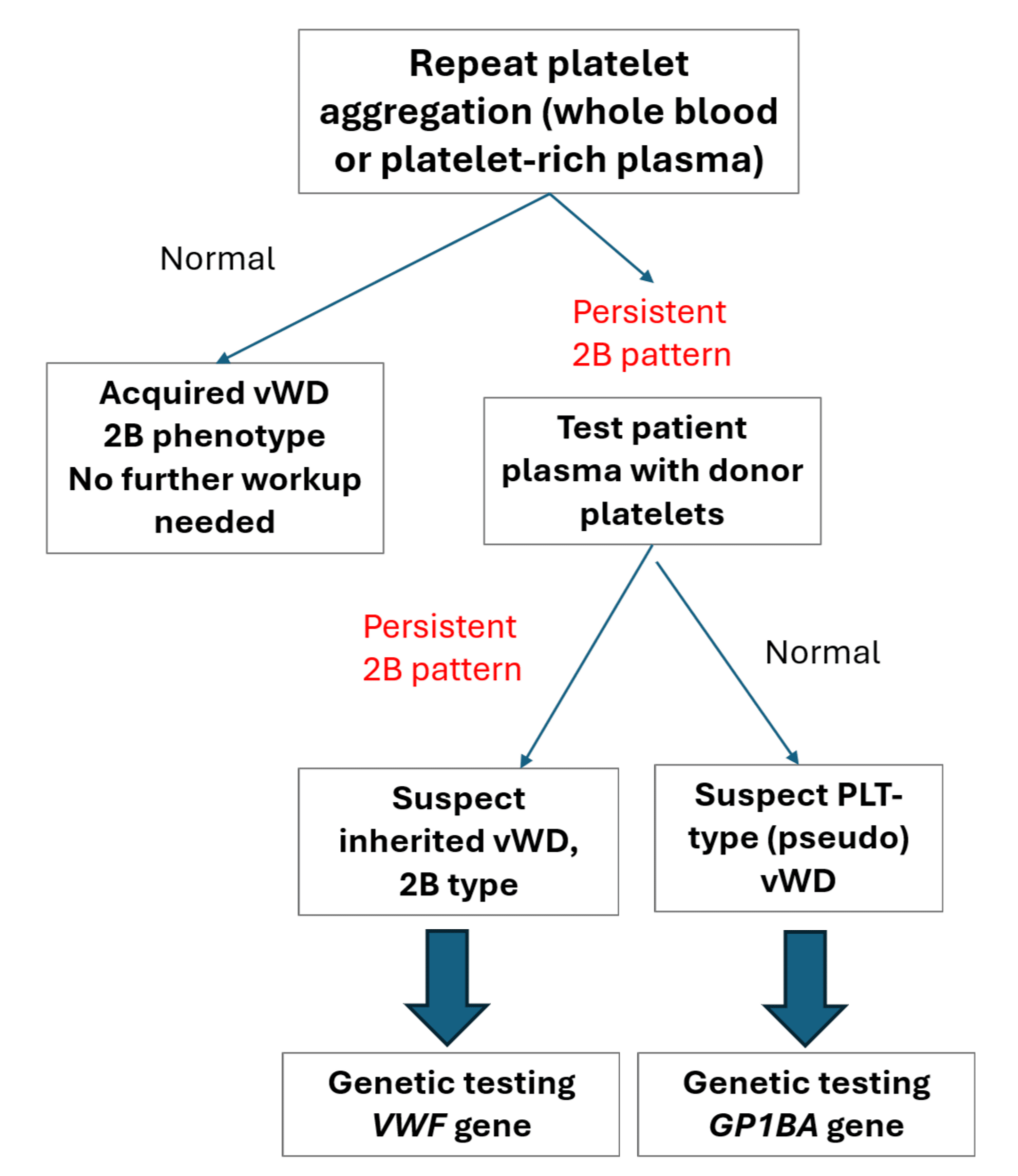
Follow-up testing algorithm with a Type 2B von Willebrand disease platelet aggregation phenotype. This figure highlights a suggested repeat testing algorithm to confirm a previously identified Type 2B vWD platelet aggregation pattern, in the presence of appropriate clinical bleeding or bruising. vWD: von Willebrand disease; PLT-type, platelet-type; vWF: gene encoding von Willebrand factor; GP1BA; gene encoding platelet receptor glycoprotein Ib

Nonetheless, we propose a triage testing algorithm (Figure 4), which we applied in our case, to guide evaluation when encountering a hyper-response abnormality to low-dose ristocetin in platelet aggregation.

While mild thrombocytopenia is often seen in Type 2B vWD, the primary differential diagnosis in our case includes acquired platelet dysfunction, which affects primary hemostasis and presents with mucocutaneous bleeding, or chemotherapy-induced thrombocytopenia. Although inherited PT vWD demonstrates identical platelet aggregation findings, a transient, acquired defect in the platelet GPIb receptor resulting in hyperaffinity has not been reported in the literature and is theoretically far less likely than a transient vWF-related defect. While bleeding has been reported in cases of severe thrombocytopenia due to rituximab therapy [19], our patient’s platelet count at the time of the abnormal platelet aggregation was near normal. Furthermore, rituximab has not been linked to causing acquired vWD and has, in some cases, shown therapeutic benefit, though results have been inconsistent [20].

## Conclusions

Acquired vWD with a Type 2B phenotype remains an extremely rare phenomenon; however, our case report uniquely describes a temporal association between acquired vWD and DLBCL with this phenotype. Our emphasis is not solely on the platelet aggregation abnormality, but rather on the challenges inherent to laboratory assay interpretation and the unique circumstances surrounding this case.

First, we believe that interference from the DLBCL itself - whether through expression of surface receptors or tumor-associated proteases - resulted in an abnormally functioning vWF, leading to bleeding/bruising and the abnormal platelet aggregation observed. Second, the presence of acute-phase reactants, tumor-related inflammation, and the patient’s advanced age obscured the interpretation of von Willebrand assays and multimer analysis. These interferences may have masked any quantifiable deficiencies, if present. Third, the normalization of platelet aggregation and resolution of symptoms following DLBCL remission supports an acquired bleeding etiology. In summary, a thorough clinical history, combined with a judicious and cautious approach to laboratory testing - while acknowledging methodological limitations - is essential in evaluating atypical presentations of vWD, including acquired forms.

## References

[1] Connell NT, Flood VH, Brignardello-Petersen R (2021). ASH ISTH NHF WFH 2021 guidelines on the management of von Willebrand disease. Blood Adv.

[2] Kumar S, Pruthi RK, Nichols WL (2002). Acquired von Willebrand disease. Mayo Clin Proc.

[3] Horiuchi H, Doman T, Kokame K, Saiki Y, Matsumoto M (2019). Acquired von Willebrand syndrome associated with cardiovascular diseases. J Atheroscler Thromb.

[4] Karger R, Weippert-Kretschmer M, Budde U, Kretschmer V (2011). Diagnosis and therapeutic management in a patient with type 2B-like acquired von Willebrand syndrome. Blood Coagul Fibrinolysis.

[5] Scepansky E, Othman M, Smith H (2014). Acquired von Willebrand syndrome with a type 2B phenotype: diagnostic and therapeutic dilemmas. Acta Haematol.

[6] Jaouen S, Mingant F, Pan-Petesch B, Lippert E, Jeanpierre E, Galinat H (2024). A rare case of acquired von Willebrand syndrome type 2B: diagnosis, treatment, and underlying pathophysiology. Res Pract Thromb Haemost.

[7] Michiels JJ, Budde U, van der Planken M, van Vliet HH, Schroyens W, Berneman Z (2001). Acquired von Willebrand syndromes: clinical features, aetiology, pathophysiology, classification and management. Best Pract Res Clin Haematol.

[8] Mahat M, Abdullah WZ, Hussin CM (2014). Conventional rapid latex agglutination in estimation of von Willebrand factor: method revisited and potential clinical applications. J Immunol Res.

[9] Ruggeri ZM (2003). Von Willebrand factor, platelets and endothelial cell interactions. J Thromb Haemost.

[10] Biguzzi E, Castelli F, Lijfering WM, Cannegieter SC, Eikenboom J, Rosendaal FR, van Hylckama Vlieg A (2021). Rise of levels of von Willebrand factor and factor VIII with age: role of genetic and acquired risk factors. Thromb Res.

[11] Sadler JE (2003). von Willebrand disease type 1: a diagnosis in search of a disease. Blood.

[12] Kawecki C, Lenting PJ, Denis CV (2017). von Willebrand factor and inflammation. J Thromb Haemost.

[13] Casonato A, Daidone V, Galletta E, Bertomoro A (2017). Type 2B von Willebrand disease with or without large multimers: a distinction of the two sides of the disorder is long overdue. PLoS One.

[14] Casonato A, Gallinaro L, Cattini MG (2010). Reduced survival of type 2B von Willebrand factor, irrespective of large multimer representation or thrombocytopenia. Haematologica.

[15] Stufano F, Boscarino M, Bucciarelli P, Baronciani L, Maino A, Cozzi G, Peyvandi F (2019). Evaluation of the utility of von Willebrand factor propeptide in the differential diagnosis of von Willebrand disease and acquired von Willebrand syndrome. Semin Thromb Hemost.

[16] Federici AB (2014). Clinical and laboratory diagnosis of VWD. Hematology Am Soc Hematol Educ Program.

[17] Szederjesi A, Baronciani L, Budde U (2020). Comparison of von Willebrand factor platelet-binding activity assays: ELISA overreads type 2B with loss of HMW multimers. J Thromb Haemost.

[18] Seidizadeh O, Eikenboom JC, Denis CV (2024). von Willebrand disease. Nat Rev Dis Primers.

[19] Jiang Y, Song J, Wang N, Yuan D, Feng L, Qu H, Fan J (2020). Rituximab-induced acute thrombocytopenia in patients with splenomegaly B Cell lymphoma: an underdiagnosed but severe complication. Cancer Biol Ther.

[20] Mazoyer E, Fain O, Dhote R, Laurian Y (2009). Is rituximab effective in acquired von Willebrand syndrome?. Br J Haematol.

